# A Novel Suture-Based Vascular Closure Device to Achieve Hemostasis after Venous or Arterial Access While Leaving Nothing behind: A Review of the Technological Assessment and Early Clinical Outcomes

**DOI:** 10.3390/jcm13164606

**Published:** 2024-08-07

**Authors:** Saami K. Yazdani, Omer Shedd, George Christy, Rex Teeslink

**Affiliations:** 1 Department of Engineering, Wake Forest University, Winston-Salem, NC 27101, USA; 2 Department of Cardiology and Electrophysiology, CaroMont Regional Medical Center, Gastonia, NC 28054, USA; omershedd@gmail.com; 3 Department of Interventional Cardiology, Advocate Good Shepherd Hospital, Barrington, IL 60010, USA; gwchristy@comcast.net; 4 EnsiteVascular LLC, Olathe, KS 66061, USA; rteeslink@ensitevascular.com

**Keywords:** vascular closure, medical device, hemostasis, venous access, large bore sheath

## Abstract

Vascular hemostasis after venous and arterial access in cardiovascular procedures remains a challenge. As sheath size gets larger for structural heart and vascular procedures, no dedicated closure devices exist that can overcome all the challenges of achieving vascular hemostasis, in particular on the venous side. Efficiently and reliably ensuring hemostasis at the access point is crucial for enhancing the safety of a procedure. Historically, hemostasis relied on manually compressing venous access sites. However, the shift towards larger sheaths and the more frequent use of continuous anticoagulation has strained this approach. Achieving hemostasis solely through compression in these scenarios demands heightened vigilance and prolonged application, resulting in increased patient discomfort and extended immobility. Consequently, manual compression may consume more time for healthcare providers and contribute to bed occupancy in hospitals. This review article summarizes the development of the SiteSeal^®^ Vascular Closure Device, a novel leave-nothing-behind approach to achieve hemostasis. The introduction of this technology has provided clinicians with a safer and more effective way to achieve immediate hemostasis, facilitate early ambulation, and enable earlier discharges with fewer access site complications compared with traditional manual compression.

## 1. Introduction

Recent advancements in percutaneous transvenous techniques for treating cardiac abnormalities, such as catheter ablations and structural heart and wireless pacemaker implantations, have transformed the landscape of cardiac care. The management approach for atrial fibrillation (AF) has notably transitioned from traditional medications and anticoagulants to procedures like ablation devices. This evolution has conversely led to a significant surge in the absolute number of procedures performed globally each year, reaching an industrial scale. Consequently, healthcare professionals are facing mounting pressure to streamline patient processing, aiming to mobilize and discharge them within hours post-procedure [1,2,3,4,5].

Several factors have contributed to the heightened importance of venous access site management in these procedures. These include the emergence of techniques necessitating larger venous sheaths, a rise in patients requiring long-term anticoagulation, and the expanding acceptance of older and more obese individuals for invasive interventions. The femoral vein is the primary choice for venous access due to its favorable characteristics, including its large size and consistent anatomy. It reliably accommodates multiple sheaths with outer diameters of up to 27 F in a single vessel [6,7,8]. In contrast, veins in the upper body are infrequently utilized. Jugular access is uncomfortable for patients, while subclavian or axillary access carries risks such as hemothorax, pneumothorax, hematomas, pseudo-aneurysms, and potential nerve damage. Additionally, the veins in the arm are often too small for the required procedures.

Traditional venous access site hemostasis has relied on manual compression, which remains the benchmark for assessing the efficacy of other hemostasis methods, especially for small-caliber venous sheaths. However, even with smaller sheaths, achieving hemostasis through compression alone can be time-consuming, taking up to 30 min, causing discomfort for patients, and placing additional burdens on medical staff. Moreover, the mandatory immobilization period of 4–8 h following manual compression adds to the cost and patient discomfort, as they need to lay flat with no mobilization. This approach also carries risks, including the potential for deep vein thrombosis and bleeding complications stemming from incomplete control or vascular injury at the access site, leading to hematoma, arteriovenous fistulae, and pseudoaneurysm formation [9,10,11,12].

Figure-of-eight (F8) suture is an alternative approach for large-bore venous-access closure, including multiple sheaths in a single vessel [13,14]. Prior studies have evaluated the safety and efficacy of the F8 suture technique through venography or vascular ultrasound. Studies have shown that F8 achieved faster hemostasis, resulting in faster ambulation and shorter overall hospital stay. Additionally, it led to significantly fewer access site complications compared to manual compression [15,16]. However, time to ambulation is still within hours (4–10), and time to discharge is typically 1 day [17,18,19]. In this paper, we describe a novel ‘leave-nothing-behind’ single vascular closure device (VCD) capable of enhancing patient mobility. The development, deployment technique, and clinical performance of this novel VCD will be discussed.

## 2. Vascular Closure Device Design Development

### 2.1. SiteSeal Vascular Closure Device

**Figure 1:**
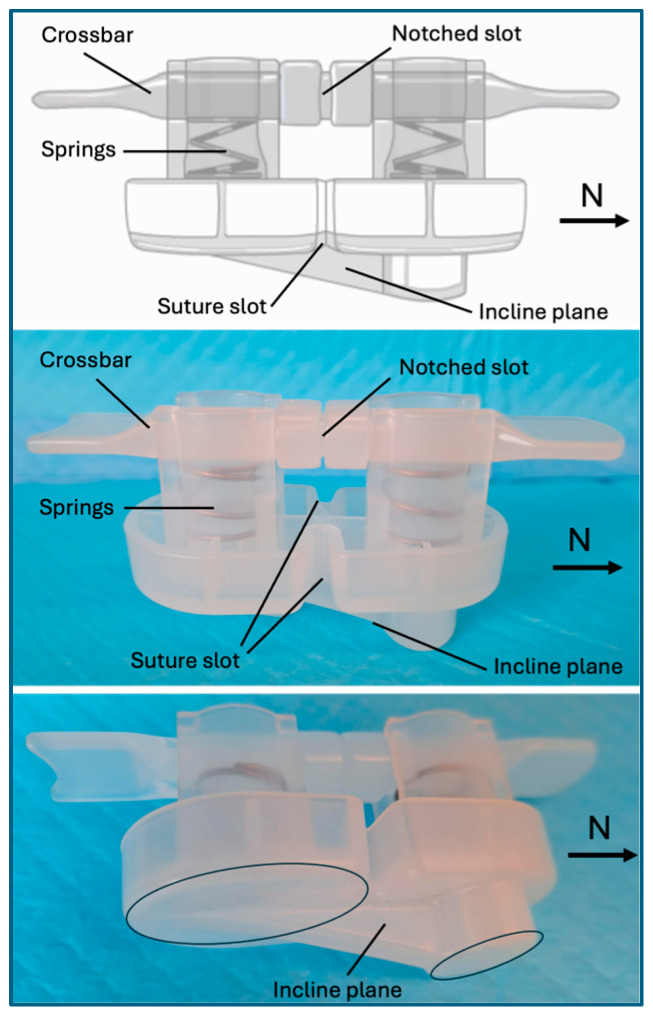
**Illustration of the SiteSeal VCD**. The SiteSeal design includes a crossbar, springs, a suture slot, a notched slot, and an incline plane. The incline plane is designed to position the north side towards the heart on the proximal side of the venotomy site.

The SiteSeal^®^ (EnsiteVascular LLC, Olathe, KS, USA) is an FDA Market cleared simple-to-use, atraumatic, vascular closure device that achieves hemostasis with minimal complications. SiteSeal is designed for every patient, regardless of shape, size, or calcification, ensuring the vessel is closed without leaving anything behind. The SiteSeal is a spring-loaded polypropylene device (Figure 1). The springs not only provide pressure during deployment to achieve hemostasis, but also help modulate heartbeat pressure variation without stopping blood flow through the vessel. Also included with the SiteSeal polypropylene device are a 2-0 Vicryl suture, hemostatic powder, a tincture of benzoin or mastisol, and elastic tape dressings for securing the device following deployment.

### 2.2. Deployment and Removal of the SiteSeal VCD

**Figure 2:**
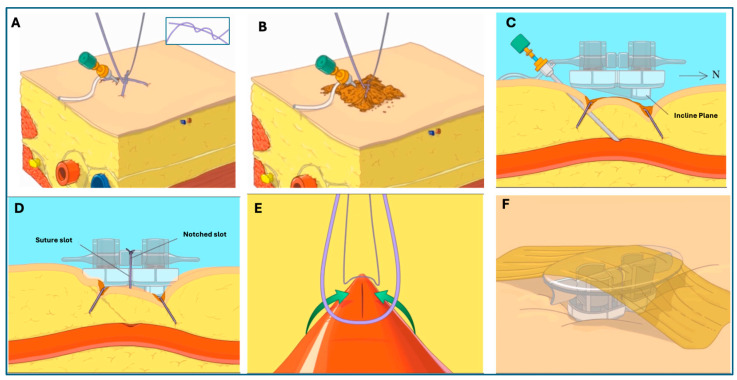
**Step-by-step deployment of the SiteSeal VCD at a venotomy site.** (**A**) A Z-stitch using a 2-0 Vicryl suture is created and a double half knot is formed, creating an ‘X’ over the venotomy site. (**B**) Hemostatic powder is then placed around the sheath and the half-knot. (**C**) The SiteSeal is then positioned over the sheath at the venotomy site with the incline plane facing north. (**D**) The sheath is then removed while the two loose suture ends are pulled tight against the sheath. The suture ends are pulled up through the design slots and tightly knotted. (**E**) Following unloading of the springs, the tension created by the Z-stitch continues to fold the soft tissue surrounding the venotomy site, closing the opening in a linear fashion. (**F**) This is followed by placing elastic tape on top of the SiteSeal device for stabilization.

SiteSeal utilizes a number 2-0 Vicryl suture to make a Z-stitch to hold the SiteSeal device in place and close the venotomy site in a linear fashion. The Z-stitch is placed by entering the soft tissue at the skin insertion site of the sheath. The first entrance is approximately 1 cm east of the sheath, passing under the sheath and exiting 1 cm west of the sheath. The second entrance for the Z-stitch is 1 cm above the skin insertion of the sheath and 1 cm to the east. The needle then crosses up and over the sheath and back down into the soft tissue, exiting 1 cm west of the sheath. The Z-stitch suture forms a double half knot which, when closed, creates an ‘X’ over the venotomy site (Figure 2A). Hemostatic powder is placed around the sheath and the half-knot to minimize oozing from the suture holes (if there are any) following suture removal (Figure 2B).

The SiteSeal device is cocked by turning the crossbar horizontally and applying pressure, which results in the loading of the springs. It is then positioned over the sheath at the venotomy site with the incline plane facing north (Figure 2C). The sheath is then removed while the two loose suture ends are pulled tight against the sheath. The suture ends are pulled up through the design slots and the suture is tightly knotted (Figure 2D). The loaded springs are then released by turning the cross-bar back to a vertical position. This action results in the pressure created by the Z-stitch to continue and fold the soft tissue surrounding the venotomy site, closing the opening in a linear fashion (Figure 2E). The roof is positioned onto the SiteSeal, and elastic tape is placed on top for stabilization (Figure 2F). An angiogram following the positioning of the SiteSeal demonstrates the closure of the venotomy site in a linear fashion.

The removal of the SiteSeal is first achieved by removing the elastic tape and then uncapping the roof of the SiteSeal. The suture between the base and the cross-bar is then cut, allowing for the removal of the SiteSeal. The next step is to remove the suture by cutting the long axis of the Z-stitch. Both ends of the Z-stitch are then removed.

## 3. Clinical Experiences with the SiteSeal^TM^ VCD

From November 2022 to April 2024, 3628 SiteSeal VCDs were placed in 2227 patients. The device has been utilized by 29 physicians at 15 different clinical sites. A total of 1464 cases included its use for electrophysiology interventions. In this subset, 424 of the 1464 patients were female (29%), with a mean age of 62 ± 11 years and 58 ± 11 years for females and males, respectively. In total, 879 of the 1464 patients (60%) were treated with three sheaths in the right common femoral vein and one sheath in the left common femoral vein. The three sheaths in the right common femoral vein consisted of two small sheaths (up to 10 Fr.) and one medium sheath (12–16 Fr.). The sheath in the left common femoral vein consisted of a medium sheath. For these patients, the left and right groin were closed with one SiteSeal device per groin. In total, 512 of the 1464 patients (35%) were treated with two sheaths (one small and one medium sheath) in the right common femoral vein and two sheaths in the left common femoral vein. For these patients, the left and right groin were closed with one SiteSeal device per groin. And, in 73 of the 1464 patients (5%), two sheaths (one small and one large; 16–27 Fr.) were positioned in the right common femoral vein. For these patients, the right groin was closed with a single SiteSeal device. Overall, in patients treated with the SiteSeal device for electrophysiology intervention, there were 0 major complications and 2.3% minor complications with zero reintervention procedures. 

In addition to electrophysiology intervention, a total of 105 cases included its use for structural heart procedures. In this subset, 49 of the 105 patients were female (47%), with a mean age of 70 ± 12 years and 73 ± 12 years for females and males, respectively. The patients were treated using one large sheath (16–27 Fr.) in the right common femoral vein and subsequently closed with a single SiteSeal device. In this cohort of patients, there were 0 major complications and 2.7% minor complications with zero reintervention procedures. 

The final cohort of patients treated with the SiteSeal devices included patients undergoing coronary artery procedures, with a total of 668 involving its use. In this subset, 200 of the 668 patients were female (30%), with a mean age of 73 ± 12 years and 75 ± 13 years for females and males, respectively. The patients were treated using one small sheath (up to 10 Fr.) in the right common femoral artery and subsequently closed with a single SiteSeal device. In this cohort of patients, there were 0 major complications and 2.5% minor complications with zero reintervention procedures. 

The overall clinical outcomes have shown a faster patient turnaround, improved patient satisfaction, the alleviation of healthcare burdens, and a lower risk of infection. These can be attributed to reducing the time to ambulation, patients’ ability to immediately elevate their head, and no restriction on leg movement following the deployment of the SiteSeal VCD. The design and delivery of the SiteSeal also leave nothing behind following positioning, reducing the risk of a foreign-body reactions and any access site complications. The SiteSeal VCD has no French size limitations within the arterial or venous systems, enabling its broad utility for physicians. And, lastly, the use of a single SiteSeal VCD can close multiple sheaths in a single vessel, as well as large bore sheaths, accelerating patient time to discharge and improving patient turnover rates with fewer complications.

In a recent single-center, prospective, published clinical study, the clinical outcome of the SiteSeal was compared to two vascular closure devices: the Vascade, a collagen-plug closure device, and Perclose, a suture-based closure device. The mean age and BMI of the patients were 68 ± 11 years and 29.7 ± 6.2 kg/m^2^, respectively. In total, 46 of the 138 patients were female (33%), and femoral vein access was obtained in all, bilaterally in 113 (82%), with a mean of 3 ± 1 access sites per patient, and a sheath size ranging from 6- to 23 Fr. Femoral arterial access was obtained in 14 (10%) cases, once per patient, with a sheath size ranging from 5- to 8- Fr. The results of this study showed no arterial access complications. On the venous side, zero (0/40) patients treated with the SiteSeal had hematomas, whereas both the Vascade group (3/57) and the Perclose group (1/41) had incidences of hematomas. In addition, the time to ambulation was less in the SiteSeal group (123 ± 11 min) compared to Vascade (139 ± 11 min) or Perclose (137 ± 34 min), although differences did not reach significance [20].

**Figure 3:**
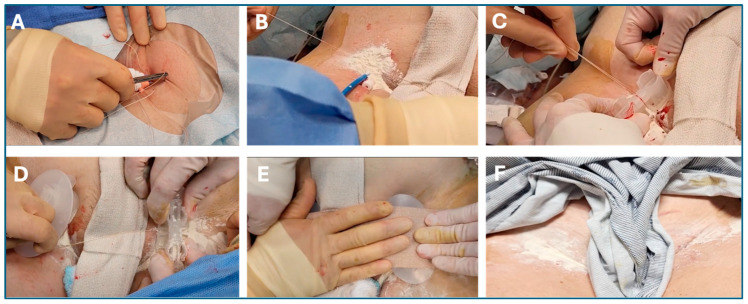
**Clinical images showing the placement of the SiteSeal VCD.** Briefly, (**A**) a Z-stitch using a 2-0 Vicryl suture is created and a double half knot is formed, creating an ‘X’ over the venotomy site. (**B**) Hemostatic powder is then placed around the sheath and the half-knot. (**C**) The SiteSeal is then positioned over the sheath at the venotomy site with the incline plane facing north. The sheath is then removed while the two loose ends of the suture are pulled tight against the sheath. The suture ends are pulled up through the design slots and the suture is tightly knotted. Following the release of the loaded springs, the roof is placed on the SiteSeal and (**D**) the procedure is repeated on the opposite groin. (**E**) This is followed by placing elastic tape on top of both SiteSeal devices for stabilization. (**F**) The removal of both SiteSeals demonstrates no hematoma or bleeding at the venotomy site.

Figure 3 shows the simple workflow of the SiteSeal placement in a clinical setting. The Z-stitch is placed, and a double-half knot is positioned at the venotomy site. Hemostatic powder is then placed at the knot and sheath location. The device is then positioned over the sheath with the incline plane facing the heart. Following the removal of the sheath, the operator pulls the suture ends tight and up through the designed slots, activating the spring mechanism. The operators then place a second device on the opposite groin, followed by placing elastic tape on top for stabilization. The tape, device, and sutures are then removed before patient ambulation.

## 4. Benefits of SiteSeal^TM^ VCD as Compared to Manual Compression and Figure-of-Eight Suture Techniques

While arterial access for percutaneous coronary interventions has shifted from predominantly femoral to predominantly radial due to equipment size reductions, the trend in transvenous interventions has been toward larger-diameter equipment. Consequently, upper limb veins are rarely adequate for these procedures. Traditional methods for achieving hemostasis at venous access sites typically involve manual compression. This approach is effective for smaller venous sheaths and serves as the benchmark for evaluating other hemostatic techniques. However, even with smaller sheaths, achieving hemostasis through compression alone can be time-consuming, often taking up to 30 min. This prolonged compression period can be uncomfortable for patients and adds to the workload of medical staff. Following manual compression, patients are typically required to remain immobilized for 4–8 h, which further contributes to the inconvenience and cost of the procedure [7,9,18,21]. Immobilization, aimed at reducing the risk of access site complications, leads to discomfort for patients, particularly in the form of back pain. Additionally, immobilization poses a well-documented risk factor for the development of deep vein thrombosis and embolism [22,23].

To overcome some limitations of MC after sheath removal to achieve post-procedure venous hemostasis, suture techniques such as figure-of-eight sutures are now widely used as an alternative. F8, along with modifications such as F8 sutures with a three-way-stopcock, has been primarily implemented to minimize the time associated with manual compression [24,25]. Several authors have highlighted that achieving an optimal workflow is among the primary challenges faced by healthcare organizations [26,27]. Turnaround time is essential for optimizing processes and ensuring patient satisfaction [26,27,28]. Improvement in workflow in the catheterization laboratory is therefore desirable. The F8 suture technique has improved the workflow compared to manual compression; however, the rate of same-day discharges remains low, at 12.3% for F8 and 3.2% for manual compression [18]. Even in the case of the modified F8, which adds a three-way stopcock, there was no change in discharge time (1.2 ± 0.4 days vs. 1.3 ± 0.6 days; *p* = 0.232) [18].

**Figure 4:**
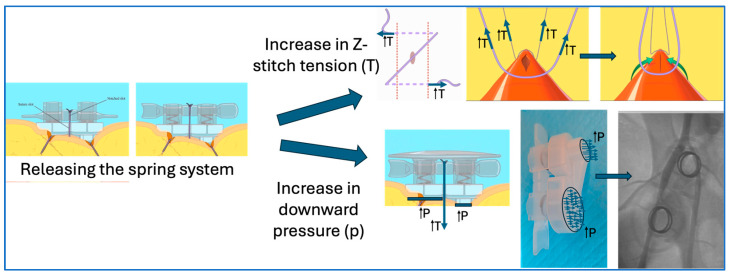
**The beneficial design of the SiteSeal spring system.** The release of the springs by the operator leads to an increase in the Z-stitch tension and increases focal downward pressure.

The main goal of the SiteSeal VCD has been to address this shortcoming and to further improve workflow, allowing for more same-day discharges. The design and implementation of the SiteSeal accelerates the hemostasis process by applying the pressure load at the venotomy site using the loaded springs, tightening the Z-stitch and ultimately closing the vascular opening in a linear fashion. This action differs to F8 or manual compression due to the combination of the Z-stitch and the SiteSeal design. Specifically, the SiteSeal is designed to allow the Z-stitch to close the venotomy site in an east–west fashion, essentially closing the venotomy in a longitudinal manner. Furthermore, the placement of the SiteSeal applies pressure on the proximal side, controlling blood flow and allowing a platelet-plug formation on top and distal to the venotomy. Additionally, the release of the springs (Figure 4) creates an increase in the Z-stitch tension, further closing the venotomy, and increases focal downward pressure. From our early clinical experiences with no major complications or the need for re-intervention, the majority of patients have been discharged on the same day. In cases where the patients were discharged the next day, none were related to the SiteSeal device, and were mainly due to patient choice (e.g., driving home late at night) or the physician’s choice due to significant associated comorbidities.

In addition to improving the overall workflow, the SiteSeal VCD was also intended to increase patient comfort. Patients treated with F8 are required to lay flat for a minimum of 2 h in a supine position, after which they can elevate the head of the bed but still must rest in a supine position for another few hours. Patients undergoing manual compression experience longer durations. Patients treated with the SiteSeal VCD, however, can immediately elevate their head to 30 degrees with no restriction on leg movement. This allows for patient comfort and minimizes back and leg pain, as they can move around. Additionally, head elevation enables patients to eat and drink immediately, decreases the chance of aspiration, and allows for more efficient breathing.

The figure-of-eight technique is also susceptible to rebleeding due to the loosening of the suture, and there is an inability to retighten the suture in the case of rebleeding [25]. Additionally, as there is no applied downward pressure, there is also potential for bleeding when the F8 is removed. The SiteSeal device crossbar design allows for the adjustment of pressure at the venotomy site. The design also enables medical staff to inspect the puncture site for bleeding or any signs of hematoma after the SiteSeal VCD has been implemented. In addition, due to the crossbar design applying the additional load onto the venotomy site, the risk of the knot snapping at the last step, as observed in F8 sutures, is minimized. Finally, since the Z-stitch is pulled up through the design slots of the SiteSeal VCD, it is easier to cut and remove. Whereas, in F8 sutures, the taut sutures often become buried in the pinched skin, making removal difficult and frequently leading to fragmentation [28,29].

It is noteworthy that other techniques, such as suture or collagen delivery devices, have also been shown to be effective for immediate femoral venous hemostasis. However, these devices are not applicable for every size of sheath (most of them approved up to 12 Fr), have significant costs because they require multiple devices for multiple sheaths in a single vessel or a large-bore sheath, and may cause clinical complications [18,30]. The SiteSeal is the only vascular closure device cleared by the FDA with no restriction to sheath size for both arterial and venous access. It can close multiple sheaths within a single vessel, as well as large bore sheaths, with one closure device for both arterial and venous access, while leaving nothing behind.

## 5. Conclusions

Effective venous hemostasis is essential for the safe performance of procedures for cardiac arrhythmias, left atrial appendage closure devices, wireless pacemakers, and other structural heart procedures. The SiteSeal VCD provides a safer and more effective way to achieve hemostasis. The SiteSeal can achieve immediate hemostasis, facilitate early ambulation, and enable earlier discharges with fewer venous access site complications compared with traditional manual compression. Furthermore, the improved workflow and efficiency are much greater compared to suture-based closing techniques, such as the F8. Along with the novel closure of the venotomy, the ability of the patient to have an elevated head and freedom of leg movement improves the overall procedure efficiency, comfort, and safety.
